# IMI 2023 Digest

**DOI:** 10.1167/iovs.64.6.7

**Published:** 2023-05-01

**Authors:** Padmaja Sankaridurg, David A. Berntsen, Mark A. Bullimore, Pauline Cho, Ian Flitcroft, Timothy J. Gawne, Kate L. Gifford, Monica Jong, Pauline Kang, Lisa A. Ostrin, Jacinto Santodomingo-Rubido, Christine Wildsoet, James S. Wolffsohn

**Affiliations:** 1 Brien Holden Vision Institute, Sydney, Australia; 2 School of Optometry and Vision Science, University of New South Wales, Sydney, Australia; 3 University of Houston, College of Optometry, Houston, Texas, United States; 4 West China Hospital, Sichuan University, Sichuan, China; 5 Eye & ENT Hospital of Fudan University, Shanghai, China; 6 Affiliated Eye Hospital of Wenzhou Medical University, Wenzhou, China; 7 Centre for Eye Research Ireland, School of Physics and Clinical and Optometric Sciences, Technological University Dublin, Dublin, Ireland; 8 Department of Ophthalmology, Children's Health Ireland at Temple Street Hospital, Dublin, Ireland; 9 Johnson & Johnson Vision, Jacksonville, Florida, United States; 10 Queensland University of Technology, Brisbane, Australia; 11 Department of Optometry and Vision Science, University of Alabama at Birmingham, Birmingham, Alabama, United States; 12 Menicon Company Limited, Nagoya, Japan; 13 UC Berkeley Wertheim School Optometry & Vision Science, Berkeley, California, United States; 14 College of Health & Life Sciences, Aston University, Birmingham, United Kingdom

**Keywords:** myopia, definitions, experimental models, interventions, ethical considerations, clinical management

## Abstract

Myopia is a dynamic and rapidly moving field, with ongoing research providing a better understanding of the etiology leading to novel myopia control strategies. In 2019, the International Myopia Institute (IMI) assembled and published a series of white papers across relevant topics and updated the evidence with a digest in 2021. Here, we summarize findings across key topics from the previous 2 years. Studies in animal models have continued to explore how wavelength and intensity of light influence eye growth and have examined new pharmacologic agents and scleral cross-linking as potential strategies for slowing myopia. In children, the term *premyopia* is gaining interest with increased attention to early implementation of myopia control. Most studies use the IMI definitions of ≤−0.5 diopters (D) for myopia and ≤−6.0 D for high myopia, although categorization and definitions for structural consequences of high myopia remain an issue. Clinical trials have demonstrated that newer spectacle lens designs incorporating multiple segments, lenslets, or diffusion optics exhibit good efficacy. Clinical considerations and factors influencing efficacy for soft multifocal contact lenses and orthokeratology are discussed. Topical atropine remains the only widely accessible pharmacologic treatment. Rebound observed with higher concentration of atropine is not evident with lower concentrations or optical interventions. Overall, myopia control treatments show little adverse effect on visual function and appear generally safe, with longer wear times and combination therapies maximizing outcomes. An emerging category of light-based therapies for children requires comprehensive safety data to enable risk versus benefit analysis. Given the success of myopia control strategies, the ethics of including a control arm in clinical trials is heavily debated. IMI recommendations for clinical trial protocols are discussed.

##  

Technological advances and innovations for managing myopia are gathering pace. Compared to 2019 when the first key International Myopia Institute (IMI) white papers were published, the needle with respect to “standard-of-care” practice has shifted significantly toward managing myopia with treatments to slow the progression of myopia.

The principal purpose of the digests is to provide a comprehensive and systematic review of the recently published evidence across the various domains in the field of myopia. The compilation and presentation of these digests rely on the experts in the field of myopia to scour and digest the vast trove of published data and provide a succinct summary of the changes in the field. Aimed at academics and practitioners as well as other interested professionals and the public, the digests aim to serve to disseminate the latest advances in the field, to serve as a concise repository, and to also provide a means of recording the progress in the field. The yearly digest 2021^1^ reviewed the advances in the field since the publication of the first set of IMI white papers in February 2019.

In this digest, the panel has identified and summarized the following key areas:

• Definitions and classification of myopia
• Experimental models of emmetropization
• Myopia control trials and instrumentation
• Interventions for controlling myopia onset and progression
• Industry guidelines and ethical considerations
• Clinical management guidelines

All sections provide updates in the field since the yearly digest of 2021 except for the industry guidelines and ethical considerations section, which reviews the field since the original IMI white paper on this topic.^2^

## IMI Digest: Definitions and Classification of Myopia

### Thresholds for Myopia, High Myopia, Hyperopia, Astigmatism, and Emmetropia

Recognizing the challenges and limitations of fixed thresholds for defining myopia, the original IMI report recommended adapting the threshold to the nature of the research and providing sensitivity analyses at different thresholds.^3^ A recent large population survey covering over a million participants described population-based prevalence figures for myopia in the city of Weifang, China.^4^ The study was conducted without cycloplegia, and myopia was defined as a spherical equivalent (SE) refraction (SER) of ≤−0.75 diopters (D). Analyses were provided at two different thresholds for myopia and high myopia, which included the IMI recommended levels of ≤−0.5 D for myopia and ≤−6.0 D for high myopia. Setting a higher threshold for myopia in noncycloplegic surveys is becoming more common. He et al.^5^ reported prevalence figures for myopia at a threshold of ≤−1.0 D on account of the lack of cycloplegia but reported the prevalence for high myopia at a threshold of ≤−5.0 D. It is well recognized that noncycloplegic studies will overestimate myopic prevalence and provide relatively more myopic refractions, especially in hyperopic individuals. These differences support adoption of a more myopic threshold for myopia but not a less myopic threshold for high myopia. Note that He et al.^5^ did also report high myopia prevalence figures at a threshold of ≤−6.0 D to “enable comparisons with previously published data.”

Changing the diagnostic thresholds of myopia to account for lack of cycloplegia is one valid approach. In the original IMI white paper, the proposed definition of myopia did not stipulate cycloplegia as a requirement but included the condition “when ocular accommodation is relaxed.” This was intentional to avoid potentially invalidating many epidemiologic studies in adults, but a large number of studies are now reporting prevalence data for myopia in children without the use of cycloplegia. In the 2021 IMI digest, we noted the growing use of a combination of the recommended SER threshold for myopia with an uncorrected visual acuity (VA) threshold in noncycloplegic studies.^1^ More sophisticated corrective analyses may further improve the validity of noncycloplegic prevalence estimates. For example, He et al.^5^ used a previously published correction formula that includes uncorrected VA, age, and noncycloplegic refraction to estimate the true cycloplegic refraction.^6^

Myopia remains the refractive error of greatest research interest at present, with other categories of refractive error receiving less attention. The aim of recommending thresholds for myopia and high myopia within the IMI white paper on defining and classifying myopia was to promote consistency in reporting and to aid study comparisons and meta-analyses. It is valid to ask if definitions for other forms of refractive error show a high level of consistency. A recent meta-analysis of population refraction data that included 41 studies with over a million participants in China reveals an interesting pattern, as shown in the Figure.^7^ Among the 41 studies, there was good consensus on the threshold values of −0.5 D for myopia and −6.0 D for high myopia, but little agreement as to whether to use ≤ or < within the definition. For hyperopia, there was clear preference for a threshold of SE ≥2.0 D, but only just over half of the surveys (23/41) actually reported a threshold or prevalence figure for hyperopia. Reports for astigmatism showed even more variability, with a threshold of SE ≥0.75 D being commonest.

**Figure.**
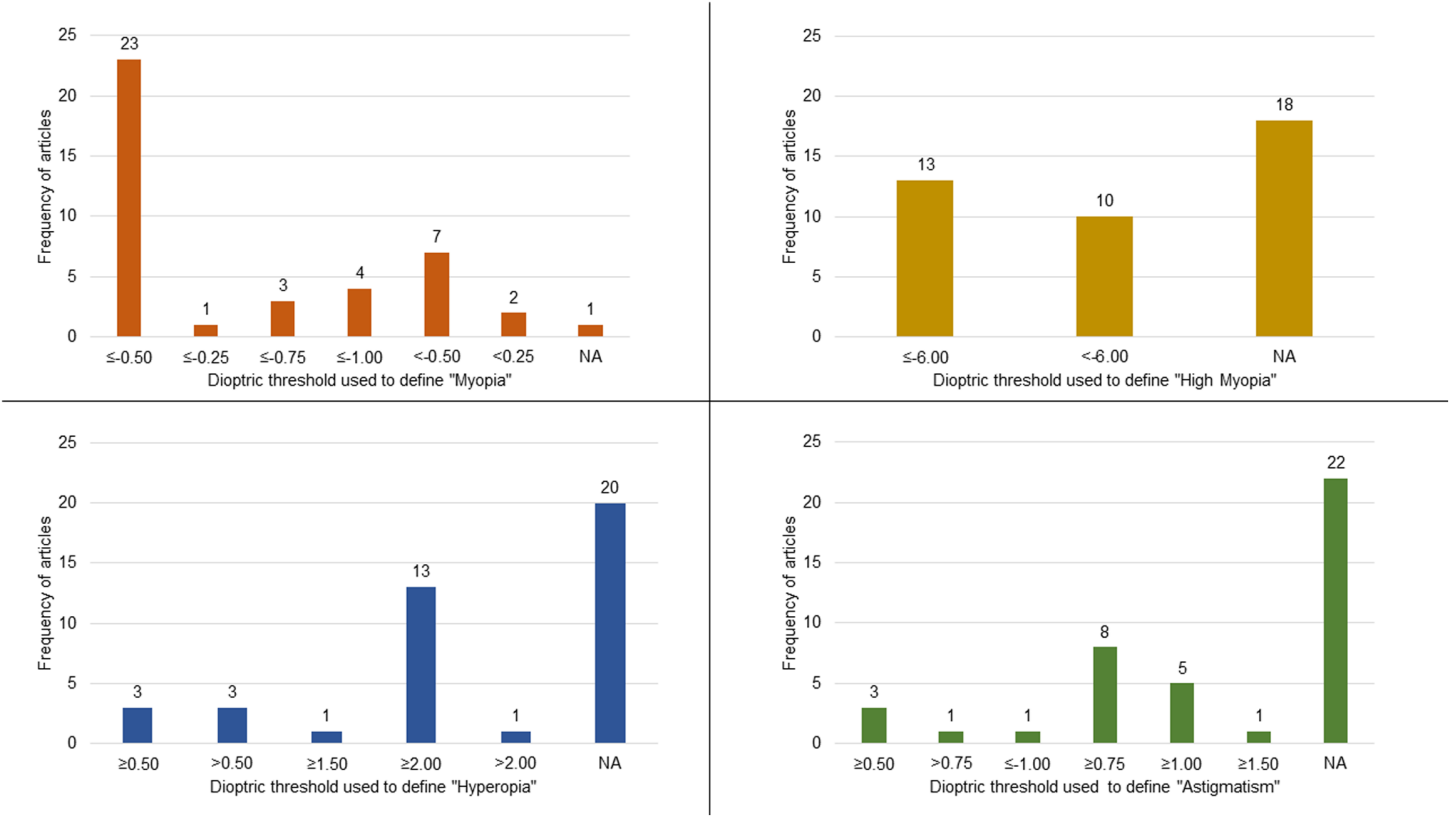
Criteria used to define myopia, high myopia, hyperopia, and astigmatism in 41 studies as extracted from Tang et al.^7^ NA, not available.

In this large collection of studies, emmetropia was defined by exclusion, as is often the case (i.e., as eyes that did not qualify as myopic or hyperopic), so the variability in the definition of emmetropia reflects the combination of the variability of the definitions for myopia and hyperopia. Although this leads to nine different numerical definitions among the 23 studies that defined both myopia and hyperopia, there is a clear consensus leader where emmetropia was defined as SE >−0.50 D and SE <2.0 D in 11 of these studies. This definition can be considered a reasonable basis for classifying functional emmetropia in children with good accommodation but not in an older population. Furthermore, it differs from the conventional optical definition of emmetropia as a refractive state of the eye where a distant object is in sharp focus on the retina when the ciliary muscle is fully relaxed. Perhaps most critically, if emmetropia is to be equated with clear distance vision, then defining it on the basis of SE ignores the detrimental visual impact of astigmatism.

The original white paper on defining and classifying myopia proposed thresholds for myopia and high myopia based on the SER while acknowledging that consideration of astigmatism and off-axis refraction may be more relevant for certain research questions. SER's dominance in epidemiologic and myopia control trials is most likely based on the statistical ease of analyzing refraction as a single scalar variable. But it is an imperfect characterization of refractive status. As noted in a recent article, considering both astigmatism and interocular differences allows for a more complete description of refractive errors.^8^ The proposal for emmetropia from the latter study provides for an absolute SE error <0.50 D and ≤0.75 D of astigmatism, which would provide for age-independent, good unaided VA. In the era of active refractive error management, emmetropia can be considered the goal of myopia prevention. When the measure of success for myopia control is achieving a final refraction and/or axial length (AL) as close to emmetropic values, the lack of consensus on defining emmetropia is all the more remarkable and merits addressing.

### Premyopia

In school-age children, refraction typically changes over several years, with slow myopic shifts in hyperopes and more rapid myopic shifts in myopes. In this age group, emmetropia can be a transient state. Indeed, as very few children are born myopic, nearly all adult myopes have transitioned through emmetropia at some point. The concept of premyopia, which was formally defined in a previous IMI white paper, addresses the dynamic nature of emmetropia in young children. In the 2021 digest, there were relatively few new publications addressing the concept of premyopia, but it has been gaining traction since and is the focus of several recent papers.^9^^–^^11^

The fact that premyopia, as determined by cycloplegic refraction, is the commonest refractive state in preschool children in Taiwan suggests that many of the factors driving the high rates of myopia in Asia are having an effect even before children start school.^9^ A similar distribution of refractive errors in primary school students has recently been demonstrated by a series of papers from China, referring to the feature as the concept of low hyperopia reserve, but it appears to be closely aligned with the concept of premyopia.^12^^–^^14^

While many studies have attempted to predict myopia onset, identifying future myopes in their premyopic phase offers the prospect of early intervention. Identification of predictive factors of progression into myopia will allow a more precise definition of premyopia. That, in turn, will help to separate young premyopes from stable emmetropic peers.^10^^,^^15^ Detailed longitudinal studies offer the best prospect of understanding the dynamics of myopia development from the premyopic phase to manifest myopia. Ideally, such studies should consider all optical components of the eye, including the crystalline lens, as demonstrated in a recent paper.^16^

As noted in the 2021 digest, there are several large ongoing trials targeting the premyopic phase with interventions such as atropine, and results from a recent small trial indicate that this represents a valid therapeutic approach, but larger and more definitive studies are required.^11^

### The Structural Consequences of High Myopia

The original IMI white paper on defining and classifying myopia noted that the terminology around the structural consequences of high myopia is by no means settled. While pathologic myopia remains a commonly used term to capture this overall concept, a range of terms is used to describe aspects of this condition. For the retinal complications, both myopic maculopathy and myopic macular degeneration are common and almost interchangeably used. A recent paper has demonstrated the challenges of creating robust definitions. Choroidal neovascularization in eyes with high myopia that do not meet the pathologic myopia definition of the META-Analysis of Pathologic Myopia Study group ended up not being classified as either age-related macular degeneration or myopic choroidal neovascularization.^17^

Recent research in the area covered by the terms *myopic maculopathy* and *myopic macular degeneration* can be largely grouped into longitudinal observational studies, anatomic/functional studies, and studies of interventions to manage myopia-related complications. While the link between these conditions and age has been well documented, valuable supportive evidence was published in 2022 regarding the interaction between age and the macular complications of myopia in both Asia and Europe.^18^^–^^21^ Modern technology such as ultra-widefield optical coherence tomography is providing new insights into the scleral complications of pathologic myopia.^22^ The role of capillary perfusion in the evolution of the macular complications of myopia remains a topic of major interest with a range of recent papers providing new insights.^23^^–^^25^ In relation to surgical treatments for pathologic myopia, the term *myopic traction maculopathy* is increasingly being used in studies and meta-analyses of surgical outcomes from vitrectomy and inner limiting membrane peeling.^26^^–^^28^

While refractive error, AL, and age remain the strongest predictors for the development of pathologic myopia, identification of other modifiable risk factors for sight loss would be very valuable. An intriguing pilot study from Singapore has demonstrated a possible autoimmune contribution, with one or more serum antiretinal autoantibodies present in all patients in this small cohort of 16 patients.^29^ Over 50% of patients were positive for anti–carbonic anhydrase II, antibodies that are more typically present in patients with cancer-associated retinopathy. Such new lines of research are important since for adult high myopes, neither their refraction nor age represent modifiable risk factors. For the large cohort of adult myopes who grew up in an era before any myopia control therapies, there is an urgent need for intervention studies that can make sight-threatening consequences of their myopia less inevitable.

### Myopia Definitions and the International Classification of Diseases, 11th Revision

At the time of writing of the original 2019 IMI white paper on myopia definitions, a submission was made to the World Health Organization (WHO) with suggestions for improving several of the definitions within the then draft version of the International Classification of Diseases, 11th Revision (ICD-11). The proposed descriptive definition for myopia (code 9D00.0) has largely been incorporated within the current definition, which now correctly attributes most myopia to axial elongation while acknowledging possible contributions from the cornea and/or crystalline lens (see new definition below). This development represents a significant improvement over the International Classification of Diseases, 10th Revision (ICD-10) definition. The ICD-11 and IMI definitions of myopia are now well aligned with minor differences in phrasing.

*ICD-11 Myopia 9D00.0:* *A refractive error in which rays of light entering the eye parallel to the optic axis are brought to a focus in front of the retina when ocular accommodation is relaxed. This usually results from the eyeball being too long from front to back, but can be caused by an overly curved cornea, a lens with increased optical power, or both. It is also called near-sightedness.*

*IMI Myopia Definition:* *A refractive error in which rays of light entering the eye parallel to the optic axis are brought to a focus in front of the retina when ocular accommodation is relaxed. This usually results from the eyeball being too long from front to back, but can be caused by an overly curved cornea and/or a lens with increased optical power. It also is called nearsightedness.*

With the advent of approved interventions to slow myopia progression, a strong case could also be made to include a code and definition for “progressive myopia.” Such a code would facilitate identification of young, progressive myopes who could potentially benefit from myopia control treatments. This could be a useful step in getting such treatments included in governmental and insurance-based health plans. There are currently no approved treatments to reduce the risk of premyopia developing into myopia, but if, as is likely, such therapies are introduced, a diagnostic code for premyopia would also be worth considering.

A suggestion was also made in 2018 to the WHO to replace the rather outdated term *degenerative myopia* that had been carried across from the ICD-10 to ICD-11 with the term *pathologic* *myopia*, which is much more widely used in clinical practice. A range of synonyms was suggested to be included, which would increase the chance of accurate encoding of such cases. The latest version of the ICD-11 coding tool (https://icd.who.int/ct11/icd11_mms/en/release) now includes an extensive list of matching terms, including pathologic myopia, which should make coding more consistent, even with the multiplicity of terms in current use. Nonetheless, at the time of preparing this digest, the term *degenerative high myopia* (code 9B76) remains the principal diagnostic term for myopic complications in the ICD-11 classification listed under “Disorders of the Retina” and is defined as follows:

*“Macular lesions occurring in people with myopia, usually high myopia, causing a decrease of the best corrected visual acuity and comprising myopic chorioretinal atrophy, myopic choroidal neovascularization and myopic retinoschisis.”*

Other pending definitions within the ICD-11 include new codes that reflect some specific retinal complications of myopia, including the following:

*9B76.1 Myopic maculopathy*

*9B76.2 Myopic traction maculopathy*

*9B76.3 Macular hole in high myopia*

*9B76.4 Retinal detachment in high myopia*

Within the ICD-11, degenerative high myopia is categorized as a retinal condition but fails to capture the sight-threatening complications of high myopia such as posterior staphyloma and myopia-associated optic neuropathy. These other additional features represents a challenge within the hierarchical structure of the ICD-11, since retinal, optic nerve, and scleral disorders are distinct categories. Therefore, degenerative high myopia may most appropriately be replaced either by the WHO-approved term *myopic macular degeneration* or *myopic maculopathy* within the existing structure. The definition could also be further updated to replace *myopic retinoschisis* with *myopic traction maculopathy* to better align it with modern usage. This would require the recognized optic nerve and scleral complications of high myopia to be placed elsewhere within the classification structure.

Optic nerve conditions such as glaucomatous optic neuropathy (9C40.9) are listed under the category of “Disorders of the Visual Pathways or Centres” within code 9C40 (disorder of the optic nerve). High myopia-associated optic neuropathy could then reasonably be placed in the same grouping as 9C40.10. While there is an existing code for scleral staphyloma (9B52), degenerative myopia (9B76) is listed as an explicit exclusion. Therefore, myopia-associated posterior staphyloma does not exist within the current classification, and a strong case can be made for a code such as 9B53 for “posterior staphyloma in high myopia.” Such a solution would provide a coherent placement of the components of pathologic myopia within the ICD-11 but leave the term itself as the overarching concept covering all the structural complications of myopia. This would also be in keeping with the perspective of the original 2019 IMI white paper on definitions and classifying myopia.

With the complexity of these issues and the range of terminology currently in use, it may be some time before a consensus is reached, but establishing a logical framework for the structural complications within the ICD-11 would be good starting point. Clear ICD-11 classifications and definitions of the various structural complications of myopia may also help to bring attention to the need for better interventions to alter the natural history of pathologic myopia.

## IMI Digest—Experimental Models of Emmetropization and Myopia

The use of animal models in the area of eye growth has been instrumental in shaping our understanding of myopia and developing treatment strategies. Animal models have allowed researchers to establish that emmetropization is an active process based on visual feedback.^30^ Expanding on this, form deprivation and defocus-induced experimental myopia have provided the framework in which to examine the influence of visual cues and pharmacologic agents on eye growth, which have, in numerous instances, translated to myopia control strategies in children. Here, the state of the field since the IMI 2021 yearly digest^1^ is reviewed and new findings on emmetropization and myopia using animal models presented.

### Signaling Pathways

With exposure to defocus, the retina generates a signaling cascade that passes through the retinal pigment epithelium and choroid to ultimately exert an effect on fibroblasts in the locally adjacent sclera. The scleral fibroblasts cause alterations of the extracellular matrix that either promote or restrain scleral remodeling and resultant changes in vitreous chamber depth. However, the chemical signals and candidate gene networks that mediate visually guided eye growth are as yet not well understood. Regarding the choroid, Summers and Martinez^31^ demonstrated in chicks that interleukin 6, a proinflammatory cytokine, may play an important role in the choroidal response in ocular growth. In the sclera, Wu et al.^32^ demonstrated that lumican overexpression contributed to form deprivation in rats by modulating the expression of TIMP-2, MMP-2, and MMP-14 and leading to scleral fibroblast apoptosis. Another recent study in chicks, which used five well-established but diverse methods of inhibiting myopia, found a universal activation of transcription factor EGR1 and downstream products, suggesting the existence of a well-defined retinal network that cannot be bypassed.^33^ Follow-up research to further characterize the signaling pathways from the retina to sclera that influence axial elongation could lead to improved understanding and new therapeutic interventions for myopia.

### Temporal Integration of Myopiagenic Stimuli

Imposed myopic blur using positive (plus) lenses consistently slows eye growth in animal models, resulting in hyperopia, while imposed hyperopic blur using negative (minus) lenses increases eye growth, resulting in myopia. The retina uses integrated visual experience over time to evaluate the magnitude and sign of defocus, with relatively brief periods of myopic blur being able to counteract relatively longer periods of hyperopic blur.^2^^,^^7^^,^^8^

 Zhu et al.^34^ recently reported that, similar to previous studies in chicks, marmosets with imposed hyperopic defocus experienced less myopic eye growth when exposed to brief periods of unrestricted vision or darkness for approximately 10% of the day. These findings demonstrate that the integration of the defocus signals for emmetropization is nonlinear in nature, with implications for myopia control strategies applied to children.

### Peripheral Retina as a Myopia Control Target

One of the more important findings using rhesus monkeys was that the fovea is not critical for emmetropization,^35^ meaning that the peripheral retina can direct eye growth. This result has had a profound influence on our understanding of emmetropization and on the design of optical lenses for myopia control. A recent study in rhesus monkeys sought to characterize the effects of eccentricity on the ability of peripheral positive power to influence refractive development.^36^ The results showed that myopic defocus in the far periphery, beyond 20° from the fovea, was not consistent in guiding refractive development. These findings underscore the importance of controlled studies in animal models for designing effective optical treatments for myopia in children.

### Pharmacologic Treatments

Based on previous studies in rhesus monkeys, as well as humans, oral 7-methylxanthine (7-mx), a metabolite of caffeine, was found effective in slowing myopia.^37^ Both caffeine and 7-mx are nonselective adenosine receptor antagonists. Caffeine can be compounded into a topical ophthalmic solution and is already a common, well-tolerated dietary element. Findings showed that indeed, topical caffeine was as effective as oral 7-mx in preventing experimental myopia in rhesus monkeys.^38^ The slowed eye growth in monkeys receiving topical caffeine was reflected by shorter vitreous chamber depth and an increased choroidal thickness. These findings suggest that topical caffeine may have potential in treatment strategies for myopia in children. However, a recent study showed that 2% topical caffeine had no positive effect in slowing the progression of myopia in Vietnamese children compared to untreated myopic children.^39^

### Circadian Rhythm, Dopamine, and Illumination Intensity

For over 50 years, it has been suggested that circadian rhythms and emmetropization are linked.^40^ Considerable research has resulted in the view that light during the day, especially high-intensity light characteristic of outdoors, activates intrinsically photosensitive retinal ganglion cells (ipRGCs), which communicate with dopaminergic amacrine cells.^41^ Dopamine is released during the day, which in turn has been shown to reduce the tendency of an eye to become myopic.^42^^,^^43^ Recent studies in chicks,^44^ guinea pigs,^45^ and mice^46^ further support a role for dopamine in emmetropization and myopia, with findings pointing to a D2-like receptor mechanism.^47^ In chicks, both levodopa (L-DOPA, a precursor to dopamine) and levodopa plus carbidopa inhibited form deprivation myopia and increased vitreal dopamine.^44^ The authors concluded that coadministration of levodopa with carbidopa may be a potential treatment for controlling myopia in children.

IpRGCs, which contain the photopigment melanopsin, project to the suprachiasmatic nucleus to relay information about environmental light and mediate circadian rhythm. A recent study used two strains of knockout mice: one that lacked melanopsin (encoded by the gene OPN4) but still had ipRGCs and one that lacked ipRGCs altogether.^48^ Findings showed that retinal dopamine signaling was reduced and myopia increased in form-deprived mice lacking melanopsin. Additionally, systemic L-DOPA treatment could partially reverse the myopia. The authors concluded that melanopsin is vital for refractive development and slowing myopia progression in mice.

The effects of reduced ambient lighting (about 50 lux) on lens-induced myopia in rhesus monkeys were also examined.^49^ While this low illumination did not directly cause myopia, it significantly reduced the ability of emmetropization to compensate for refractive errors, such as recovering from myopia. These findings are in accordance with previous studies suggesting that at least some exposure to high-intensity illumination is critical for optimal refractive development.^50^

### Longitudinal Chromatic Aberration

Virtually all vertebrate camera-type eyes have significant longitudinal chromatic aberration (LCA): short wavelengths focus in front of longer wavelengths.^51^ LCA would thus seem to be an ideal visual cue for emmetropization, because both the magnitude and the sign of defocus could be inferred from the relative sharpness of retinal images between shorter and longer wavelengths. However, some early results suggested that experimental animals could emmetropize under spectrally narrowband light that would not provide LCA cues for defocus.^52^^–^^54^ Because the shorter-wavelength cones are typically spatially sparse and seemingly unable to judge defocus accurately enough to achieve emmetropia, LCA was not thought to be critical for emmetropization. However, more recent results indicate that chromatic cues for defocus are generally essential for accurate emmetropization,^55^ and optical modeling has demonstrated that the spatial distribution of short-wavelength cones is adequate for using longitudinal chromatic aberration to accurately guide emmetropization.^56^^,^^57^ A recent study using tree shrews demonstrated that a video display with a chromatic simulation of myopic blur could overcome a myopiagenic environment, further demonstrating the great potency of chromatic cues for emmetropization.^58^

### Narrowband Ambient Illumination

While emmetropization typically operates under spectrally broadband illumination, given the apparent importance of chromatic cues, it should come as no surprise that drastic alterations to the ambient spectrum—in particular, narrowband light—can have profound effects on emmetropization.

#### Narrowband Long Wavelength Light – “Red” and “Amber”

Long-wavelength red light produces a powerful and consistent hyperopic/antimyopiagenic effect in both tree shrews^59^^–^^61^ and rhesus monkeys.^62^^,^^63^ More recently, it was shown that amber light, spanning a relatively broad band of frequencies but omitting those shorter than 500 nm, also promotes hyperopia in tree shrews.^64^ However, long-wavelength light has not been consistently found to slow eye growth in the other common animal models of myopia, and in fact, red light rearing induces eye growth in chickens,^54^^,^^65^^,^^66^ guinea pigs,^67^^–^^70^ and fish.^71^^,^^72^ Red light has not been widely studied in mice, although one study did suggest that red light promotes hyperopia in this species.^73^ There is currently no sufficient explanation for the divergence of the effects of long-wavelength ambient lighting between species, and it remains a major puzzle in the field. Nevertheless, findings from tree shrews and rhesus monkeys have recently been translated to short-duration red light therapy as myopia control in children.^74^^,^^75^ Further testing in animal models is necessary to understand dose–response effects and the underlying mechanism of action of long-wavelength light on eye growth.

#### Narrowband Short Wavelength Light – “Blue” and “Violet”

Blue and violet light consist of shorter wavelengths with higher energy than red and amber light. Studies have reported that blue light rearing in chicks slows form deprivation myopia^76^ and, in guinea pigs, slows defocus-induced myopia, potentially through a retinoic acid–related mechanism.^77^ On the other hand, blue light rearing in tree shrews tends to dysregulate eye growth, ultimately leading to variable refractive errors ranging from hyperopia to myopia.^78^

More recently, attention has turned to violet light. Violet light, with wavelengths between about 360 and 400 nm, is largely absent from our indoor environments and blocked by most spectacle and contact lenses. Additionally, the ocular media filter out most light of this wavelength in humans.^79^ It has been proposed that lack of violet light could be myopiagenic and that adding violet light back could be useful in preventing myopia.^80^ Currently, the majority of research on violet light has been in the mouse. The finding of a novel opsin neuropsin, or OPN5, with an absorbance peak of about 385 nm,^81^ alongside human clinical trials suggesting that violet light could indeed be antimyopiagenic,^82^ has resulted in increased interest in investigating violet light in animal models. A recent paper on mice showed that violet light suppresses lens-induced myopia via an OPN5-mediated mechanism.^83^ Again based on data from mice, it has been suggested that the intrinsic circadian rhythms of the retina are mediated specifically by violet light and OPN5,^84^ although this interpretation has been disputed.^85^ Activity in this area continues to increase, and it is only a matter of time before more data from other animal models become available, most critically from diurnal mammals with ocular media absorption properties close to the human.

### ON Versus OFF Pathways

All classical photoreceptors (rods and cones) hyperpolarize upon exposure to light. At the first synapse, the retina generates ON and OFF pathways, which are important for detecting luminance increments and decrements. Evidence suggests that emmetropization depends more on the ON than the OFF pathway,^86^^,^^87^ and work exploring this topic using animal models continues. In further support of the idea that the ON pathway is more generally important for retinal processing than OFF, a recent paper on mice suggested that ON pathway disruption results in greater deficits in visual function and dopamine signaling than OFF pathway disruption.^88^ Note that while both short- and long-wavelength sensitive cones have dedicated ON bipolar cells, the short-wavelength cones are lacking an OFF bipolar.^89^ Thus, it would be possible to calculate a short- versus long-wavelength chromatic signal using only the ON pathway but not using only the OFF pathway (or, at least, not as easily). Conceivably, the relative importance of the ON versus OFF pathway could be due to the reliance of emmetropization on short- versus long-wavelength contrast. Further research into this area could yield insight into the specific retinal circuits driving emmetropization.

### Scleral Cross-linking

With increasing age, emmetropization ultimately ceases, possibly because the accumulation of natural cross-links between collagen fibers renders the sclera relatively fixed.^90^ It has therefore been proposed that accelerating collagen cross-linking in the sclera could be an effective means of myopia control.^91^ A recent study in tree shrews using retrobulbar injections of the crosslinking agent genipin did show effectiveness against form deprivation myopia^92^ but was associated with significant retinal pathology.^93^ Pathologic changes were also seen with scleral cross-linking in the guinea pig.^94^ Previous studies in rabbits have suggested that using a blue light–riboflavin combination to induce scleral cross-linking can increase scleral stiffness with no pathologic effects.^95^ While another recent study in rhesus monkeys also suggested this method to be relatively benign, to date, the effectiveness of this approach in slowing myopia has not been demonstrated.^96^ Scleral cross-linking could, in principle, be an effective means of myopia control, but safe techniques of inducing scleral cross-linking must be developed.

## IMI Digest—Clinical Myopia Control Trials and Instrumentation

The 2019 IMI Clinical Myopia Control Trials and Instrumentation report^97^ reviewed the evidence from existing myopia control trials of at least 1 year in duration, along with supporting academic literature. The IMI 2021 yearly digest updated this evidence.^1^ These reviews provided informed recommendations on the design of future clinical trials to demonstrate the effectiveness of treatments at slowing myopia progression and the impact of these treatments on patients. Relevant publications since then up until September 2022 include 5 studies on spectacle interventions^98^^–^^101^ (1 on part-time single vision wear),^102^ 4 soft contact lens (SCL) trials,^103^^–^^106^ 1 orthokeratology (ortho-k) study,^107^ 10 studies examining the effect of prescribing atropine (although only half assessed efficacy in a prospective trial),^108^^–^^117^ and trials of atropine combined with either ortho-k^118^ or SCL^119^ or auricular acupoint stimulation.^120^ The number of prospective clinical trials on myopia control has risen substantially. As of 2018, there were 25 trials,^97^ with an additional 12 trials from 2018 to 2020^1^ and a further 26 trials from 2020 to 2022. In addition, there were two retrospective trials involving rigid corneal lenses^121^ and atropine,^122^ but they are not included in this report.

### Participant Selection Criteria

The recent trials have mainly used cycloplegic refraction, with participant selection criteria for maximum astigmatism ranging from –0.75 D to –2.50 D (generally lower for optical intervention studies) and for maximum anisometropia ranging from 1.00 to 2.00 D (although not reported in several studies), minimum distance VA from 16/20 to 20/40 (although more use 20/25 than the previously recommended 20/20, with one study stating that the number of logMAR letters read which will differ between charts with different maximum size letters presented),^107^ a minimum age from 3 to 9 years, and a maximum age typically between 10 to 16 years (Table 1). Recruiting patients with high astigmatism and anisometropia makes it more difficult to evaluate the effects of any myopia intervention as they are likely to have a very different optical environment (such as an increased depth of focus).^123^ Thus, it is recommended to exclude those patients unless it is the focus of the research. The progression of childhood myopia slows with age. Therefore, recruiting older children up to 16 years of age in a trial that lasts several years may reduce the apparent effectiveness of the intervention when considering the actual reduction in eye growth in millimeters or diopters. On the other hand, if treatment effects are reported as a percentage reduction, then as highlighted previously,^1^^,^^124^ enrolling older children could yield a falsely higher increased apparent efficacy. Enrolling older children also adds complexities, for example, the necessity of additional exclusion criteria such as “negative pregnancy test for females with childbearing potential” in new investigational device/pharmaceutical trials. Based on the evidence, none of the criteria, other than the minimum VA, appear to warrant revision from the previously recommended participant selection criteria. Thus, the updated participant selection criteria are as follows:

**Table 1.** Selection Criteria in Prospective Recent Myopia Control Clinical Trials

Author, Year	Intervention	SER (Min to Max), D	Cycloplegia	Ast Limit, D	Aniso Limit, D	VA Min	Age, Min to Max, y
Bao et al. 2022^101^	Spectacles (highly aspherical lenslets or slightly aspherical lenslets)	−4.75 to −0.75	Y	1.50	1.00	20/22 (0.05 logMAR)	8 to 13
Chamberlain et al. 2022^103^	SCL (concentric dual zone)	−4.00 to −0.75	Y	0.75	1.00	20/25	8 to 12
Chan et al. 2022^108^	0.01% atropine	−5.00 to −0.50	Y	1.00	NR	20/20	7 to 10
Chuang et al. 2021^109^	0.05% atropine (concentration increased if >0.50 D 6-month progression)	−0.50 to −5.00	Y	2.00	NR	NR	NR (figures show ∼5 to 8)
Cui et al. 2021^110^	0.02% or 0.01% atropine	−6.00 to −1.25	Y	2.00	1.00	16/20 (20/25)	6 to 14
Fu et al. 2021^111^	0.01% or 0.02% atropine	−6.00 to −1.25	Y	2.00	1.00	20/25	6 to 14
Han et al. 2021^125^	Auricular acupressure	−0.50 to +0.50	Y	NR	NR	20/25 (from decimal)	8 to 9
Hieda et al. 2021^104^	SCL (multifocal)	−6.00 to −1.00	Y	1.50	1.50	20/20	Grades 1 to 6 in school (Japan)
Hieda et al. 2021^112^	0.01% atropine	−6.00 to −1.00	Y	1.50	1.50	20/20	6 to 12
Jakobsen and Moller 2022^107^	ortho-k	−4.75 to −0.50 (sphere)	Y	2.50	1.50	78 ETDRS letters	6 to 12
Jiang et al. 2022^74^	Low-level red light therapy	−5.00 to −1.00	Y	2.50	1.50	20/20	8 to 13
Jones et al. 2022^119^	0.01% atropine with multifocal SCL	−5.00 to −0.50 (spherical component)	Y	1.00	2.00	20/25	7 to 11
Kong et al. 2021^120^	0.01% atropine plus auricular acupoint stimulation	−6.00 to +0.50	Y	1.50	1.50	NR	7 to 12
Lam et al. 2021^98^	Spectacle (novel plus powered multiple segments)	−5.00 to −1.00	Y	1.50	1.50	20/20	8 to 13
Mori et al. 2021^82^	Violet light transmitting spectacles	−4.40 to −1.50	Y	1.50	1.50	NR	6 to 12
Moriche-Carretero et al. 2021^113^	0.01% atropine	−4.50 to −0.50	Y	1.50	1.00	20/30	5 to 11
Prousali et al. 2022^102^	Part-time single-vision spectacles	−6.00 to −0.50	Y	1.50	1.50	20/25	4 to 16
Rappon et al. 2022^99^	Spectacles (two novel diffusion optics patterns)	−4.50 to −0.75	Y	1.25	1.50	20/25	6 to10
Ruiz-Pomeda et al. 2021^105^	SCL (concentric dual zone)	−4.00 to −0.75	Y	1.00	1.00	20/25	8 to 12
Saxena et al. 2021^114^	0.01% atropine	−6.00 to −0.50	Y	1.50	1.00	20/40	6 to 14
Shen et al. 2022^106^	SCL (extended depth of focus)	−8.00 to −1.00	Y	1.75	2.00	20/25	9 to 14
Wang et al. 2022^115^	0.02% or 0.01% atropine	−6.00 to −1.25	Y	2.00	1.00	20/25	6 to 14
Weng et al. 2022^117^	SCL (extended depth of focus and concentric dual zone)	−3.50 to −0.75	Y	0.75	0.75	20/32	7 to 13
Yam et al. 2022^116^	Atropine (0.01%, 0.025%, 0.05%)	−1.00 or more myopic refraction	Y	2.50	NR	NR	4 to 12
Yuan et al. 2021^118^	ortho-k with 0.01% atropine	−4.00 to −1.00	N	1.50	NR	25/25 stated	8 to 12
Zhu et al. 2022^100^	Customized PALs	−4.00 to −0.50	Y	1.50	1.00	20/20	7 to 14

Ast, astigmatism; Aniso, anisometropia; ETDRS, early treatment diabetic retinopathy study; N, no/none; NR, not reported; PAL, progessive addition lens; Y, yes.

Inclusion Criteria

**Table:** 

Refractive error:	Cyclopleged spherical or SE myopia of at least –0.75 D in each eye
	Astigmatism: ≤1.00 D in each eye
	Anisometropia: ≤1.50 D
Age:	6 to 12 years
Visual acuity:	0.10 logMAR in each eye

Exclusion Criteria

**Table:** 

History:	Previous rigid lens wear or myopia control treatment
Ocular disease:	Any (other than myopia)
Binocular vision:	Anomaly (strabismus)
Systemic disease:	Those that may affect vision, vision development, or the treatment modality
Medications:	Those that may affect pupil size, accommodation, or have an impact on the ocular tissue

Departure from these criteria will generally lead to the apparent efficacy of a treatment being under- or overestimated, with differences in approaches making it more difficult to compare across studies.^124^ It is noted that the inclusion criteria for age, VA, astigmatism, and anisometropia have widened more recently, as reflected in the recent publications of myopia control studies.

### Study Design

While previously, most studies followed their cohort for 2 years, with an additional year in some studies to examine for any rebound, study results are being published earlier^74^^,^^102^^,^^106^^,^^111^^,^^114^^,^^120^^,^^125^ and/or becoming more complex, sometimes involving longer durations with a “crossover” for the control group^103^ or longer tracking for faster-progressing myopia^109^ (Table 2). However, all studies still show a reduced effectiveness of treatments in the second year, demonstrating the need for more than 1 year of follow-up to adequately assess the long-term efficacy of the treatment. The extrapolation of a 1-year treatment effect to multiple years (an approach taken by many myopia calculators, for example) can lead to incorrect conclusions,^124^ and hence the prior IMI recommendation for a clinical trial assessing the efficacy of a treatment for myopia control of a 3-year minimum study duration (at least 2 years of treatment plus an additional year of no treatment to examine any rebound effect) is still upheld. More recent studies have not applied randomization,^109^^,^^119^ while others that randomize participants have not appropriately applied masking,^102^^,^^105^^,^^107^^,^^113^ and stratification of participants has also become more common.^99^^,^^104^^,^^105^^,^^112^^,^^116^^,^^118^^,^^120^ Several recent studies did not have a control group^109^^,^^111^ while others used historical controls.^98^^,^^119^ Nonetheless, appropriate controls were selected for the comparison group in most studies, and in the case of ortho-k and rigid corneal lens use, which cannot be easily masked, single-vision spectacles (SVSs) were used as controls.^107^ However, some atropine studies^110^^,^^115^ and one SCL study^105^ used a SVS control (no drops), and thus participants would not have been masked and compliance potentially altered. In addition, many studies made no attempt at a true sham for their control group,^74^^,^^82^^,^^113^^,^^120^^,^^125^ thus increasing the potential for bias.

**Table 2.** Control Group, Randomization, and Masking of Recent Myopia Control Trials

Author, Year	Intervention	Control	Randomization	Stratification	Masking	Study Length, y	Rebound Assessment
Bao et al. 2022^101^	Spectacles (highly aspherical lenslets or slightly aspherical lenslets)	Spectacle (SV)	Y	N	Y	2	N
Chamberlain et al. 2022^103^	SCL (concentric dual zone)	SCL (SV)	Y^†^	N	Y	6^†^	Y (planned)
Chan et al. 2022^108^	0.01% atropine	Vehicle drop	Y	N	Y	1.5	N
Chuang et al. 2021^109^	0.05% atropine (concentration increased if >0.50 six-month progression)	N	N	N	N	10	N
Cui et al. 2021^110^	0.02% or 0.01% atropine	No treatment Self-selected	Y/N (if self-selected atropine, randomized to 0.01% or 0.02%)	N	Y (if in an atropine arm)	2	N
Fu et al. 2021^111^	0.01% or 0.02% atropine	N	Y (to 0.01% or 0.02% atropine)	N	Y	1	N
Han et al. 2021^125^	Auricular acupressure	No treatment	Y	N	Y (examiner only, not participant)	1	N
Hieda et al. 2021^104^	SCL (multifocal)	SCL (SV)	Y	Y (adaptive randomization)	Y	2	N
Hieda et al. 2021^112^	0.01% atropine	Vehicle drop	Y	Y	Y	2	N
Jakobsen and Moller 2022^107^	OK	Spectacle (SV)	Y	N	N	1.5	N
Jiang et al. 2022^74^	Low-level red light therapy	No treatment	Y	N	Y (examiner only, not participant)	1	N
Jones et al. 2022^119^	0.01% atropine with multifocal SCL	Historical controls (multifocal SCL group and SV SCL group)	N	N	N	3	N
Kong et al. 2021^120^	0.01% atropine plus auricular acupoint stimulation	0.01% atropine	Y	Y	Y (examiner only, not participant)	0.5	N
Lam et al. 2021^98^	Spectacle (novel plus powered multiple segments)	Historical controls	Y	N	Y	2^*ǂ*^ (original control group followed 1 year on treatment)	N
Mori et al. 2021^82^	Violet light transmitting spectacles	Spectacle (SV)	Y	Y	Y	2	N
Moriche-Carretero et al. 2021^113^	0.01% atropine	No treatment	Y	N	N	2	N
Prousali et al. 2022^102^	Part-time spectacle SV	Full-time spectacle SV	Y	N	N	1	N
Rappon et al. 2022^99^	Spectacles (two novel diffusion optics patterns)	Spectacle (SV with tint)	Y	Y	Y	3 (reported 1-year results)	N
Ruiz-Pomeda et al. 2021^105^	SCL (concentric dual zone)	Spectacle (SV)	Y	Y	N	3	Y (last year of study)
Saxena et al. 2021^114^	0.01% atropine	Vehicle drop	Y	N	Y	1	N
Shen et al. 2022^106^	SCL (extended depth of focus)	SCL (SV; contralateral eye)	Y	N	Y	1	N
Wang et al. 2022^115^	0.02% or 0.01% atropine	No treatment	Y (to 0.01% or 0.02% only if chose atropine instead of spectacles)	N	Y (only in atropine arms)	2	N
Weng et al. 2022 ^117^	SCL (extended depth of focus and concentric dual zone)	SCL (SV; contralateral eye)	Y	N	Y	1	N
Yam et al. 2022^116^	Atropine (0.01%, 0.025%, 0.05%)	Vehicle drop (year 1 only)	Y	Y	Y	3	Y (study year 3, half of each group)
Yuan et al. 2021^118^	ortho-k with 0.01% Atropine	ortho-k with vehicle drop	Y	Y	Y	2	N
Zhu et al. 2022^100^	Customized PALs	Spectacle (SV)	Y	N	Y	2	N

† Followed for an additional period after ending randomization.

ǂ Randomized first 2 years, then all wore intervention lenses for an additional year with original control group compared to historical control group matched on age and refractive error.

The ethical dilemma of including a control group in studies^1^ remains unresolved despite the increasing evidence for the effectiveness of various myopia control treatments. Terminating a treatment to investigate the possibility of rebound presents a similar ethical issue. As also highlighted in the section on industry guidelines and ethical considerations, several studies have suffered from a high control group dropout (particularly if participants are not well masked) and more difficult recruitment due to parents not wanting their child to risk receiving the placebo. An alternative is use of historical controls as applied by two recent studies,^98^^,^^119^ but careful matching for important covariates such as age, sex, season (for shorter studies), refractive error, AL, environmental exposure, parental myopia, and race/ethnicity is required. Other alternatives include comparison with a gold standard, although equivalence analysis has statistical challenges that need to be carefully considered,^126^ or survival analysis,^127^ such as the time taken for participants’ myopia to progress more than –0.50 D, allowing participants on placebo to exit early while other treatments should still be beneficial, although this approach precludes the assessment of efficacy over longer time periods. Multisite studies generally include a larger number of participants, a population cohort recruited from wider geographic locations, and the ability to compare results across study sites, all of which increase the generalizability of the study results, but so far, they are rare.^99^^,^^128^

### Outcome Measures

The outcomes of myopia progression clinical trials can still be classified as primary (refractive error and/or AL), secondary (patient-reported outcomes and treatment compliance), and exploratory (peripheral refraction, accommodative changes, ocular alignment, pupil size, outdoor activity/lighting levels, anterior and posterior segment imaging, and tissue biomechanics).^97^ Visual disturbances (subjectively reported symptoms such as halos and glare or measured objectively such as with halometry) and patient- reported outcomes were added to the minimum data requirements for different modalities of treatment in the 2021 digest.^129^ In addition, choroidal thickness has been added as a recommended key exploratory and possibly predictive^130^^,^^131^ measurement for all forms of myopia control (Table 3; also see IMI 2023 white paper on choroid).^132^

**Table 3.** Expected Minimum Data for Each Treatment Modality

Treatment Modality	Distance Visual Acuity	Near Visual Acuity	Contrast Sensitivity^*^	Pupil Size	Cycloplegic Refraction	Choroidal Thickness^*^	Axial Length	Amplitude of Accommodation^*^	Visual Disturbances	Lens Centration^*^	Wearing Time	Instillation Compliance	Patient-Reported Outcomes
Spectacles	X	X	X	X	X	X	X	X	X		X		X
Soft multifocal contact lenses	X	X	X	X	X	X	X	X	X	X	X		X
Orthokeratology	X	X	X	X	X	X	X	X	X	X	X		X
Pharmaceuticals	X	X	X	X	X	X	X	X	X			X	X

* Indicates at least in a powered subgroup.

The 2021 digest advocated reporting of both a percentage and an absolute amount of reduced myopia progression/axial elongation in future clinical trial reports,^129^ and this was supported by a position paper published shortly after.^124^ Percentages can be misleading and so should never be reported alone. Ninety-five percent confidence intervals should also be reported along with the magnitude of change. Subgroup analysis must be planned *a priori* along with appropriate statistical power/sample size calculations. Any *post hoc* subgroup analyses should be clearly identified as exploratory and, as such, should be used for forming new hypotheses rather than proof of efficacy.

### Instrumentation

There has been a focus on imaging and segmenting (particularly using machine learning) choroidal thickness using A-scan (traditionally termed *biometers*)^133^ and B-scan (termed *optical* *coherence* *tomography*)^131^^,^^134^^,^^135^ based techniques to better understand the mechanism(s) of myopia control and also as a potential predictor of long-term efficacy of treatments (see IMI 2023 white paper on choroid).^132^ A model using principally baseline pupil area, 1-month change of the zone 3-mm (flat) and zone 5-mm (flat/steep) keratometry was able to predict between 54% and 63% of the variation in 1-year AL elongation with ortho-k.^136^ In addition, models have been developed to predict cycloplegic refractive error from demographics, noncycloplegic SER, AL/corneal curvature radius ratio, uncorrected VA, and intraocular pressure, with the results explaining 92% to 93% of the variability in Chinese school children (aged 5–18 years)^137^^,^^138^ and 96% in children in Japan (aged 2–9 years).^139^

## IMI Digest: Interventions for Controlling Onset and Progression of Myopia

Interventions to slow myopia progression are increasingly becoming the “standard of care,” with the use of specialty optical products also steadily climbing.^140^ Furthermore, combination or multimodal interventions (for example, ortho-k with low-concentration atropine) are being explored, with the goal of improving efficacy. The evidence since the original IMI white paper^141^ and the IMI yearly digest 2021^1^ was reviewed, and this update is largely limited to results from recent prospective, randomized clinical or group-matched trials, with a focus on SE refractive error and/or AL data as key outcome measures of efficacy and rebound effects on treatment discontinuation. Not comprehensively reviewed are data covering aspects such as visual performance and safety; data from studies that do not conform with standard clinical trial designs (e.g., see various studies^102^^,^^142^^–^^146^) were also not comprehensively reviewed.

### Spectacle Lens Designs

Novel spectacle lens designs represent a significant growth area with respect to myopia control. New performance data for the defocus incorporated multiple segments (DIMS) lens, which was covered in the 2021 digest, are now available. Specifically, at the end of year 2 of the trial, some of the control group were switched from SVS lenses to DIMS lenses and tracked for another year along with the DIMS group, with children in both groups being approximately 10 years old. Switching from the control to DIMS group led to significantly reduced progression compared to that over the previous year (i.e., change in SE/AL of –0.52 ± 0.69 D/0.31 ± 0.26 mm as compared to –0.92 ± 0.81 D/0.57 ± 0.33 mm, respectively) and comparable to the progression of the DIMS group in year 1.^98^ Note, however, that age represents a potential confounder in the latter comparison.

Two-year clinical trial data are now also available for two other closely related spectacle lens designs incorporating either slightly or highly aspherical lenslets in their peripheries (SAL and HAL, respectively) (Table 4).^101^^,^^147^ Children aged 8 to 14 years were randomly assigned to wear SAL, HAL, or SVS lenses. Myopia progression was significantly reduced with both lenses, with the HAL lens outperforming the SAL lens.^147^ The myopia control effects evident at the end of year 1, indexed by unadjusted mean changes in SER and AL, were enduring; at the end of 2 years, progression remained significantly reduced with HAL and SAL lenses as compared to SVS lenses: –0.66 (0.09), –1.04 (0.06), and –1.46 (0.09) D for SER and 0.34 (0.03), 0.51 (0.04), and 0.69 (0.04) mm for AL in the HAL, SAL, and SVS lens groups, respectively.^101^

**Table 4.** Summary of Key Study Design Features and Outcomes for Myopia Intervention Trials Involving Novel Spectacle or Soft Contact Lenses

Author, Year	Country	Sample Size	Lenses	Length, y	% Loss to Follow-up/Discontinued	Mean Change in SE (D)/AL (mm)	% Slowing Myopia Progression	% Slowing Axial Elongation	Age Range, y	Baseline Age, y	Myopia Range (D)	Average Myopia (D)
**Spectacle lens designs**												
Bao et al. year 1^147^	China	167	HAL and SAL vs. SV spectacles	1	3.4	HAL: −0.30/0.14 SAL: −0.48/0.24 SV: −0.79/0.35	HAL: 63 SAL: 40	HAL: 61 SAL: 31	8–13	HAL: 10.7 ± 0.2 SAL: 10.1 ± 0.2 SV: 10.4 ± 0.2	−0.75 to −4.75	HAL: −2.70 ± 0.14 SAL: −2.31 ± 0.13 SV: −2.46 ± 0.12
Bao et al. year 2^101^	China	167	HAL and SAL vs. SV spectacles	2	7.8	HAL: −0.68/0.35 SAL: −1.04/0.50 SV: −1.45/0.68	HAL: 55 SAL: 28.8	HAL: 51 SAL: 26	8–13	HAL: 10.7 ± 0.2 SAL: 10.1 ± 0.2 SV: 10.4 ± 0.2	−0.75 to −4.75	HAL: −2.70 ± 0.14 SAL: −2.31 ± 0.13 SV: −2.46 ± 0.12
Rappon et al. 2022^99^	USA	258	Test 1 and test 2 vs. SV spectacles	1	9.3	Test 1: −0.68/0.35 Test 2L: −1.04/0.50 SV: −1.45/0.68	Test 1: 74 Test 2: 59	Test 1: 50 Test 2: 33	6–10	Test 1: 8.0 ± 1.2 Test 2: 8.2 ± 1.2 SV: 8.2 ± 1.2	−0.75 to −4.50D	Test 1: −2.00 ± 0.93 SAL: −1.85 ± 0.91 SV: −1.95 ± 1.02
**Contact lens designs**												
Shen et al. 2022^106^	Taiwan	72	Contralateral center near EDOF vs. SV	1	6.9	EDOF: −0.70/0.34 SV: −0.88/0.38	20.5	10.5	9–14	12.4 ± 1.5	−1.00 to −8.00	EDOF: −3.31 ± 1.26 SV: −3.32 ± 1.17
Weng et al. 2022^117^	China	95	Group I: Bilateral SV Group II: Contralateral SV vs. EDOF Group III: Contralateral SV vs. MiSight	Stages 1 and 2: 0.5/0.5 Stages 1 and 2: 0.5/0.5 Stages 1 and 2: 0.5/0.5	33.3 48.4 50.0	Refer to article as multiple stages/lens combinations	— 39 and 64 41 and 30	— 63 and 66 48 and 42	7–13	10.9 ± 1.5 10.8 ± 1.5 10.8 ± 1.6	−0.75 to −3.5	Group I: −2.08 ± 0.64 Group II: −2.01 ± 0.64 Group III: −1.91 ± 0.72
Fang et al. 2022^160^	China	81	Group I: SVCL Group II: ortho-k Group III: MFSCL	1	3.8 17.2 7.7	SVCL: −1.00/0.45 ortho-k: NA/0.34 MFSCL: −0.63/0.31	— ortho-K: NA MFSCL: 37.2	— ortho-K: 31.1 MFSCL: 24.4	7–15	13.0 ± 0.2 12.5 ± 0.2 12.8 ± 0.1	−1.00 to −8.00	Group I: −3.00 ± 0.28 Group II: −2.66 ± 0.21 Group III: −3.14 ± 0.30
Jones et al. 2022^119^	USA	138	MFSCL (+2.50 D) + 0.01% atropine MFSCL (+2.50 D) SVCL	3	Age matched		52.2 49.5 —	42.6 54.4 —	7–11	10.4 10.1^*^ 10.2^*^	−0.75 to −5.00	−2.21 ± 0.80 −2.31 ± 1.00 −2.31 ± 0.89
Garcia-del Valle et al. 2021^149^	Spain	70	Reverse geometry SCL vs. SVCL	1	17.1	Test CL: −0.28/0.13 SVCL: −0.57/0.22	51	41	7–15	12.2 ± 2.2 11.9 ± 2.1	−0.50 to −8.75	−2.80 ± 1.79 −3.31 ± 1.76

CL, contact lens.

* Median.

Year 1 data are also now available for a 3-year trial of another novel spectacle lens design incorporating diffusion optics technology, which are intended to reduce spatial contrast (only a small, ∼ 5-mm, central area of the lens is free of diffusing elements). In this trial, children aged 6 to 10 years were randomly assigned to wear either a lightly tinted SVS lens (control) or one of the two test lenses, which varied in the density of dots, with test 1 lens having a lower dot density compared to test 2 lens. Year 1 results suggest robust myopia control effects, with the apparently greater response with the test 1 lens. Mean changes in SER/AL were –0.14 D/0.15 mm and –0.22 D/0.20 mm with test 1 and test 2 lenses compared to –0.54 D/0.30 mm with the SVS lens.^99^

### SCL Designs

Recent clinical trials confirmed that myopia control strategies are effective in older children. In an extension of a 3-year trial of dual-focus compared to single vision SCL (SV SCLs), a subset of participants who were approximately 13 years of age were continued in dual-focus SCLs for a further 3 years, over which period myopia progression was found to slow significantly in those switched from SV SCLs to dual-focus SCLs and the treatment effect sustained in those continuing with dual-focus SCLs.^103^

In a 12-month randomized, contralateral, crossover clinical trial involving bilateral SV SCLs, contralateral SV versus extended depth of focus (EDOF) SCLs, and contralateral SV versus MiSight SCLs (lens assignments randomized by eye in contralateral groups), both EDOF and MiSight SCLs were found to slow myopia progression as compared to SV SCLs, with similar efficacy.^148^ Participants were aged 7 to 13 years. In another, similarly designed trial, a center-near EDOF SCL was found to significantly slow myopia progression relative to that with an SV SCL over 12 months,^106^ although the treatment effects (i.e., 0.18 D/0.04 mm) are small compared to findings with other lens designs.

The list of myopia control contact lens (CL) designs continues to grow. A custom-made, lathe-cut SCL with a peripheral progressive +2.00 D add on the front surface is one such design. While details on this design are scant, its reverse geometry design is intended to aid in lens stability, while providing continuous peripheral defocus.^149^ In a randomized trial of this SCL involving children aged 7 to 15 years, myopia progression was reduced by approximately 50% over 12 months (i.e., mean change in SE/AL of –0.28 D/0.13 mm compared to –0.57 D/0.22 mm for the control group).

### Orthokeratology

Novel (ortho-k) lens designs have been the subject of some recent, mostly small-scale, trials. In one such trial involving a contralateral design, significantly different increases in AL were reported after 18 months for a multifocal ortho-k lens (center-distance, +2.50 D) compared to a conventional ortho-k lens, consistent with superior efficacy of the former.^150^ That myopia control can be enhanced by reducing the back optic zone diameter (BOZD) of ortho-k lenses is the shared conclusion of two additional studies. In one study,^151^ AL increases over 12 months were significantly less with a 5 mm compared with a 6 mm BOZD lens (by 0.72 mm) (Table 5), with the difference in the treatment zone also positively correlated with the AL change. The second study, which involved young adolescents (13.34 ± 1.38 years of age), reported a 0.13-mm absolute reduction in AL elongation over 12 months.^152^ The compression factor represents a third ortho-k design feature examined in this context, with a compression factor of 1.75 D versus 0.75 D with new versus conventional ortho-k lenses (i.e., an additional 1.00 D compression factor) linked to slower axial elongation, by 34% (0.35 ± 0.29 mm vs. 0.53 ± 0.29 mm) after 2 years of lens wear.^153^

**Table 5.** Axial Elongation in Millimeters Reported in the Various Studies Involving Orthokeratology

Author, Year	Lenses	Eye	12 Months	18 Months	24 Months
Guo et al. 2021^151^	OK (BOZD 6 mm) (*n* = 32)		0.17 ± 0.13		
	OK (BOZD 5 mm) (*n* = 26)		0.04 ± 0.15		
Lau et al. 2022^153^	OK (*n* = 29)^*^				0.53 ± 0.29
	OK (IC) (*n* = 35)				0.35 ± 0.29
Loertscher et al. 2021 (*n* = 28)^150^	OK (1 eye)			0.129	
	OK (MF) (the other eye)			−0.044	
Long et al. 2020^154^					
Unilateral anisometropes	SV spectacles (*n* = 38)	NME	0.31 ± 0.32		
		ME	0.33 ± 0.29		
	OK (*n* = 79)	NME^†^	0.34 ± 0.21		
		ME	0.05 ± 0.19		
Bilateral anisometropes	SV spectacles (*n* = 37)	LME	0.35 ± 0.28		
		MME	0.38 ± 0.21		
	OK (*n* = 98)	LME	0.15 ± 0.19		
		MME	0.05 ± 0.17		
Tan et al. 2022^166^	OK (*n* = 35)				0.35 ± 0.20
	OK (A) (*n* = 34)				0.17 ± 0.19

A, 0.01% atropine; IC, increased compression by 1 D; LME, less myopic eye; ME, myopic eye; MME, more myopic eye; NME, nonmyopic eye.

* Lens parameters were not modified to correct significant residual myopia (if any), to maintain 1 D difference in compression factor between the two groups of subjects.

† No lens wear.

The efficacy of ortho-k as an intervention for controlling anisometropia has also been recently explored,^154^^–^^158^ with the unanimous conclusion that it is an effective treatment. Specifically, AL elongation slowed more in the more myopic eyes, effectively reducing the anisometropia. Interestingly and potentially indirectly related, in another study, 20 subjects who were initially categorized as slow progressors based on their rates of axial elongation showed no clinically significant change 7 months after being switched from SVS to ortho-k, whereas 21 of 24 subjects identified as fast progressors did.^159^

In another comparative efficacy trial, aspheric multifocal soft contact lenses (MFSCLs, +6.00 D max. peripheral power), ortho-k, and SVS lenses were included in a 1-year single-blind, randomized clinical trial.^160^ The MFSCLs and ortho-k showed similar efficacy; AL changes, used as indices of progression, were 0.30 and 0.31 mm, respectively, compared to 0.41 mm with SVS lenses.^160^

### Pharmaceutical Interventions

#### Oral 7-Methylxanthine

This adenosine antagonist, a close relative of caffeine, is already approved for use in Denmark, the location of a recent observational study,^161^ in which data from a patient cohort of 7 to 15 years (<–0.50 D or worse myopia; *n* = 711) were used to examine the effects of age and dose (range, 400–1200 mg). The main conclusion was that dose counts—the highest dose offered the best control.

#### Topical Atropine

Topical atropine remains the only widely accessible ophthalmic formulation with an established efficacy profile, but differences in formulating the composition may likely affect the outcome.^162^ Recent data indicate that age matters, at least in Chinese children, with younger children requiring higher concentrations to achieve similar reductions in myopia progression^110^^,^^163^ (e.g., 0.05% vs. 0.025% for 6- vs. 8-year-olds).^110^ That concentration and age influence rebound responses were well demonstrated in year 3 data of the LAMP study, which compared washout (no therapy) versus continued therapy (0.05%, 0.025%, and 0.01%). Those previously receiving higher concentrations exhibited faster (rebound) progression, with differences between washout and continued therapy groups being smaller for older age groups.^116^ In one of two other studies of interest, monocular 0.125% atropine therapy, administered to the longer eye of anisometropes, was found effective in reducing interocular differences by slowing axial elongation.^164^ The second study, a small trial involving premyopic children (4–12 years old) and 0.01% atropine, indicated that topical atropine may delay the onset of myopia.^165^

Trials combining topical atropine and myopia control optical interventions have yielded mixed results. In a 3-year trial involving MFSCL with a +2.50 D add,^119^ the addition of daily 0.01% atropine did not lead to improved efficacy. However, in another randomized clinical trial involving children assigned to either an ortho-k alone (OK) group or a combined 0.01% atropine-ortho-k (AOK) group,^166^ significantly slower axial elongation was found in the AOK group compared to the OK group at the end of the 2-year trial. Slower axial elongation was also associated with a larger increase in the photopic pupil size, potentially implicating increased higher-order aberrations as a source of directional defocus cues.^166^

### Rebound Effects—Relative Risks for Optical Versus Pharmaceutical Interventions

Rebound phenomena, as observed after the termination of long-term use of pharmacologic agents, are of debatable relevance to optical interventions. It is thus not surprising that progression after discontinuation of MiSight SCLs was similar to progression with SVSs, albeit in a small sample.^105^ Likewise in a contralateral trial comparing progression with EDOF and MiSight SCLs and contralateral SV SCLs, no “rebound” was observed after discontinuation of the two “myopia control” SCLs.^117^

### Light Therapies

 Since 2021, there have been four publications reporting results from clinical trials involving low-level red light (LLRL) therapy and a single retrospective study.^74^^,^^75^^,^^167^^–^^169^ In all cases, two 3-minute direct exposures to LLRL per day, spaced at least 4 hours apart, were provided via desktop, long-wavelength (635–650 nm) laser diode devices, with energy outputs in a range of 0.29 to 0.4 mW. In some but not all cases, therapy was limited to weekdays, with participation largely limited to children, down to 3 years of age in one retrospective study.^75^ Only one short (6-month) trial included a sham treatment, in the form of a dimmer red light treatment (0.03 compared to 0.29 mW).^167^ Across trials, the longest follow-up period was 2 years,^168^ with change in AL/SE with LLRL being 0.16 ± 0.37 mm/–0.31 ± 0.79 D as compared to 0.64 ± 0.29 mm/–1.24 ± 0.63 D with SVS alone. While the large treatment effects, which are generally greater in magnitude than those reported with other pharmacologic and optical interventions, are attracting much attention, there remain important issues related to these trial data that need to be addressed. Consistent across all studies was an early (detectable within first month) AL shrinkage in a large number of eyes, a parallel reduction in myopia (i.e., hyperopic shift),^74^^,^^168^ and choroidal thickening, contrasting with the choroidal thinning in control groups.^74^^,^^75^^,^^168^^,^^169^ However, the mechanism underlying this AL shrinkage remains uncertain and cannot be explained by changes in choroidal thickness. Likewise, why are there only modest rebound effects on termination of the LLRL therapy?^168^^,^^169^ The safety of such LLRL therapies remains to be established, as none of the trials to date have included suitably sensitive objective functional testing and plans for long-term follow-up. Additionally, adverse event monitoring has been largely questionnaire based, with passing reference to optical coherence tomography (OCT) imaging in certain studies.^74^^,^^168^^,^^169^

#### Violet Light and Myopia Control

In the only clinical trial to date,^82^ myopia progression was tracked in 6- to 12-year-old children assigned to either violet light (360–400 nm) transmitting spectacles or conventional spectacle lenses. The treatment effect of the violet light lenses proved to be small and not significant (0.03-mm and 0.11-D slowing over 2 years with relative reduction in axial elongation by 21.4%).

## IMI Digest: Industry Guidelines and Ethical Considerations

Since the original IMI report on industry guidelines and ethical considerations,^2^ there have been significant advancements and developments in the field of myopia control with an increasing adoption of myopia control strategies by eye care practitioners worldwide. This digest updates on the findings since the last report and expands on areas of recently acquired knowledge.

### Safety

The original 2019 IMI report on industry guidelines and ethical considerations^2^ asserted that “children do not have a higher risk than adults of suffering from contact lens-related complications with either OK or soft contact lens wear”. Recent studies have largely supported this assertion.^170^^–^^174^ While spectacle lenses present no risk with regard to infection, they may affect vision. Thus, it is important to note that new novel spectacle lenses specifically designed for myopia control have some influence on visual performance (measured using high- and low-contrast visual acuity, reading speed, and peripheral contrast sensitivity),^175^^–^^177^ although the use of contact lenses has been shown to improve how children and teenagers feel about their appearance and participation in activities, leading to greater satisfaction with their refractive error correction.^178^^–^^181^ Atropine can cause cycloplegia and photophobia at higher concentrations,^182^ requiring the supply of photochromic progressive addition lenses, although they are not necessary at lower concentrations.^183^ As discussed in the “Light Therapies” section, at the present time, there is a lack of comprehensive data and review of safety with the newly emerging light therapies.

### Efficacy

The 2019 IMI report on industry guidelines and ethical considerations^2^ noted that both refractive error and AL can be used to assess the efficacy of myopia control—the former ideally using cycloplegic autorefraction to minimize patient and examiner biases and the latter using optical biometers because of their exquisite precision. A compelling case for axial elongation being the preferred primary outcome^124^ is based on its stronger relation to visual impairment,^184^ superior precision,^185^^,^^186^ and its immunity to accommodation artifacts.^187^ Most important, some myopia control modalities, primarily overnight ortho-k,^188^ modify corneal shape, thus affecting the refractive status of the eye and making refraction measures untenable for assessing myopia progression.^124^

Myopia control efficacy is usually assessed by comparing annual refractive error progression, axial elongation, or both between treated and untreated myopic children. An important observation is that the efficacy in the first year of treatment is generally greater than in subsequent years,^124^ a feature that appears to be true for both optical and pharmaceutical modalities. This comparison of mean progression is also the primary outcome in US Food and Drug Administration (FDA) clinical trials of devices, be they contact lenses or spectacles.^128^^,^^189^ Investigational drugs are evaluated by a different unit of the FDA, where the favored primary outcome appears to be the overall between-group difference in proportion of subjects who show a given difference in myopia progression after 3 years, either –0.50 or –0.75 D.

The rigorous requirements for FDA approval include assessment of safety and patient-reported outcomes, as well as evaluation for potential rebound effect, as has been demonstrated in some,^190^^–^^192^ though not all,^116^^,^^193^^,^^194^ studies involving myopia control interventions.

### Worldwide Regulatory Status of Modalities

The term *myopia management* is used by eye care practitioners and optometric associations worldwide to broadly refer to clinical strategies used by eye care practitioners to address a patient's immediate refractive error condition, namely, correcting their myopia, as well as assessing the progression of their condition over time (i.e., reducing myopia progression) and axial elongation. This terminology does not distinguish, however, between products specifically approved for *myopia control* (on-label) from those only approved for the correction or temporary reduction of myopia—although the latter may slow myopia progression in children (off-label). Typically, regulatory approval is required to ensure that medical products meet certain standards of safety and efficacy before being authorized for use. When prescribing a treatment for myopia control, where possible, the eye care professional should ideally start by considering on-label products and contemplate off-label prescribing when on-label products are not effective or appropriate.^2^

At the time of the original 2019 IMI reports, there appeared to be only two products that had regulatory clearance anywhere in the world—both were multizone SCL lenses that were Conformité Européenne (CE) marked, which is the manufacturer's self-certification that the products conform to the standards within Europe. Since then, several soft and ortho-k contact lenses have obtained CE-marked approval specifically for reducing myopia progression in children, which not only permits commercialization of these products for this indication within the European Union but also facilitates pursuing marketing authorization for myopia control in other parts of the world, such as Australia and Singapore. Of special interest is that the FDA has approved the first SCL (MiSight; Coopervision Inc., Pleasanton, California, USA) specifically for myopia control. Although the FDA makes a distinction between *myopia control*—an indication reserved for devices slowing myopia progression—and the broader term of *myopia management*, the two terms are used interchangeably by eye care practitioners and optometric associations both within and outside the United States.

Low-concentration atropine eye drops appear to be commercially available for slowing myopia progression in children in parts of Asia, notably Singapore. Also, a proprietary solution of 0.01% atropine eye drops has recently obtained regulatory approval in Australia to slow the progression of myopia in children (https://www.nps.org.au/medicine-finder/eikance-0-01). In other countries, including the United States, low concentrations of atropine are increasingly used off-label but must be compounded with variations in procedures and thus formulations.^162^

The regulatory approval process varies around the world in its scope and rigor. The FDA typically requires 3-year data from a controlled randomized clinical trial, with 1-year follow-up after termination of treatment to assess the potential for rebound effects,^189^ while other jurisdictions may accept shorter-term studies or other forms of evidence. For example, regulatory agencies in parts of Asia may base their positions on those of the FDA, and discussion of its stances here is germane to other regions. The range of products that have been approved for slowing myopia progression and are now marketed in different countries has grown dramatically since the 2019 IMI reports, and their number and diversity are expected to continue to expand. Thus, any attempt to document approved products by region would likely be incomplete and, very soon, obsolete.

### Dissemination of Information

The 2019 IMI report on industry guidelines and ethical considerations^2^ states, “One major issue relates to the fact that myopia control treatments do not impart an immediate effect but rather an expected outcome that is several years in the future.” Recent work has quantified the long-term reduction in years of visual impairment that might be expected from a program of myopia control and placed it in the context of the short-term risks.^195^ Nonetheless, some bodies feel that long-term visual benefits in adult life accrued as a result of myopia control in childhood should be confirmed by prospective studies (College of Optometrists Myopia Management, https://www.college-optometrists.org/the-college/policy/myopia-management.html, accessed January 2023), despite the 50 to 60 years that such an enterprise would take. Furthermore, the potential benefits of myopia control should be placed in the context of its cost.^196^

### Consideration of Location of Studies

The 2019 IMI report on industry guidelines and ethical considerations^2^ opined that “previous studies have shown that a given treatment (e.g., ortho-k) might not present the same efficacy in clinical trials conducted in different countries and ethnic groups.” Most clinical trials are single-center studies with limited ethnic and racial diversity. Multicenter trials are rare and multicountry studies even rarer.^99^^,^^128^ Nonetheless, a growing body of evidence shows that overnight ortho-k slows axial elongation to a similar degree in East Asian^197^^–^^201^ and non–East Asian^107^^,^^202^^–^^204^ populations, with studies conducted in diverse locations across the world also reporting remarkably similar treatment effects.^205^ Likewise, progressive addition spectacles lenses are equally ineffective in East Asian^206^^–^^208^ and non–East Asian^193^^,^^209^^,^^210^ populations. Finally, low-concentration atropine has limited efficacy across East Asian, South Asian, and Australian populations,^110^^,^^112^^,^^114^^,^^183^^,^^211^^,^^212^ although underpowered analyses of small subgroups suggest differences may exist.^212^

### Ethical Issues of the Future of Clinical Trials in Myopia Control

The IMI 2021 Digest^129^ questions, “If the treatment is well enough established to slow or prevent myopia progression, is it ethical to randomly assign subjects to an ineffective sham/control group given their likelihood to develop myopia or have myopic progression?” The conclusion was that “at present, an appropriately selected concurrent control group is still ethical for myopia control trials.” There are concerns about the ethics of removing a successful myopia control treatment from a participant. As the evidence for the relation between myopia level and visual impairment grows,^184^^,^^195^ along with that for effective therapies for slowing progression, the above question should be visited periodically. There are additional, practical challenges to the conduct of clinical trials where an effective treatment is withheld in control subjects in order to evaluate a new therapy. As summarized in the “Study Design” section and a recent study,^213^ these include parents immediately withdrawing a child assigned to a no-treatment group^192^^,^^214^ and a higher proportion of longer-term withdrawals among control subjects.^212^^,^^215^ Ultimately, ethical questions are best answered region by region, with the availability of on-label myopia control modalities and the prevailing standard of care being key considerations. Alternative clinical trial designs have been summarized in the “Study Design” section,^213^ including using a virtual control group based on previous studies,^216^ comparison with established treatments, or a time-to-treatment-failure (survival analysis) approach.^217^

## IMI Digest: Clinical Management Guidelines

This digest supplements the 2019 IMI white paper on clinical management guidelines^218^ and the IMI 2021 yearly digest^1^ by providing a broad update on the previous IMI publications. For specific updates on new treatments for myopia, the reader is referred to the previous section on interventions for controlling myopia onset and progression. In this section, new information on the comparative efficacy, safety, and visual outcomes of myopia control interventions was extracted from relevant publications and collated to inform and guide clinical practice. Clinical considerations, including balancing short-term risks and long-term benefits, are also explored.

### New Understanding of Treatments and Efficacy

#### Comparative Treatment Efficacy

A recent review of myopia control treatments highlighted gaps in current myopia research.^124^ One key posit was the concept of Cumulative Absolute Reduction in Axial Elongation (CARE), proposed as an alternative to “percentage efficacy” in evaluating treatments and when comparing treatments outside of a single study. Within a study, percentage efficacy represents the percentage reduction in myopia progression in the treatment compared to the control group. However, as the treatment outcome is influenced by many factors, such as study duration and participant characteristics, which may vary significantly between clinical trials, percentage efficacies cannot be validly compared across studies. Instead, it was recommended that myopia control treatment efficacy be reported as an absolute value—the total reduction in axial growth of the treatment group compared to the control group. This paper was the first to compare absolute efficacy outcomes for a variety of spectacle, MFSCL, and ortho-k studies, all of which included at least 10 data points. While pharmacologic interventions were not included due to concentration-dependent efficacies, there was no apparent superior treatment, with “the best of ortho-k, MFSCLs, spectacles and atropine showing similar effect.” Further support for this statement was provided in recent clinical trials, which found similar myopia control efficacy between MFSCLs and ortho-k compared to the control group,^160^ as well as between MFSCLs and EDOF lenses.^117^

While some myopia control treatments may be less effective, and both side effects and the potential for rebound can impact outcomes, eye care practitioners should factor in their own skill set, treatment availability of treatments, patient and parent preferences and capacity, and, finally, regulatory considerations when choosing a treatment for the individual patient.^124^

Although the concept of CARE has the potential to further expand our understanding of myopia control treatment efficacy, eye care practitioners need to compare patient outcomes with a control reference group, which can vary based on age, ethnicity, gender, and parental myopia, to determine if a patient is experiencing lower than expected cumulative absolute progression, indexed by axial elongation. Furthermore, CARE does not consider variations in treatment effect or a proportional treatment outcome (i.e., where children showing faster progression prior to treatment experience a larger myopia control effect), as was shown in one 6-year study.^103^ Despite the ongoing debate over reporting absolute versus proportional treatment effect, proactive treatment of all young myopes, especially those under age 12 years, is recommended.^124^

#### Maximizing Outcomes: Wearing Time

Research supports wearing time and/or treatment compliance as a potential avenue to explore to maximize treatment outcomes, with data indicating that full-time wear of myopia control treatment achieves the best outcome. For HAL spectacles, myopia control efficacy was highest in children who wore their HAL spectacles for at least 12 hours per day, 7 days per week.^101^ This observation also aligns with results from previous MFSCL trials that found increased efficacy with improved wearing time, measured in hours per day^219^ or days per week.^217^ Other studies of dual-focus SCLs^128^ and DIMS spectacle lenses^220^ have reported similar benefits with longer wearing times.

#### Maximizing Outcomes: Combination and Enhanced Treatments

Combination strategies offer another method to further improve the efficacy of existing myopia control treatments. However, as indicated in the previous section on interventions for controlling onset and progression of myopia, outcomes have been mixed with some demonstrating a benefit,^166^ while others report no added benefit over monotherapy.^119^ It would be of interest to determine if efficacy can be enhanced by combining atropine with myopia control spectacle lenses or by using higher concentrations of atropine in combination treatments. However, a retrospective study found that combining ortho-k with a higher concentration of atropine (0.125%) was not as effective as a lower concentration (0.025%) of atropine.^221^ This result is seemingly paradoxical, given the concentration-dependent results of several atropine-only studies,^110^^,^^183^^,^^222^ although only one trial to date has directly compared concentrations above and below 0.1%.^222^

Modifying ortho-k lens designs may also influence treatment effect. As outlined in the section on interventions, wear of ortho-k lenses with 6-mm compared to a 5-mm BOZD resulted in slower axial elongation in the latter case, after 1 year of wear, despite the lenses having a slightly lower first-fit success rate. However, the difference in axial elongation of 0.12 mm was significantly different between groups only in the first 6 months, with axial elongation continuing at a similar rate in both groups during the second 6-month period.^151^

#### Rebound of Myopia

A review of myopia control efficacy stated that “rebound should be assumed until proven otherwise.”^223^ In this respect, although the aggregate of data as summarized here and in the previous section on interventions indicates minimal rebound upon cessation of current myopia control SCL and low-concentration atropine treatments, discontinuation of treatment before 13 to 14 years of age should be undertaken with caution. Specifically, no rebound was observed when children aged 13.2 ± 1.2 years discontinued dual-focus SCL wear after 2 years of lens wear.^105^ Similarly, discontinuation of low-concentration atropine (0.025% and 0.01%) after 2 years of treatment did not result in any significant rebound effects, while a faster eye growth was observed after discontinuation from 0.05% atropine (0.04 mm over 1 year), although deemed to be clinically insignificant. Also, in 6- to 8-year-old children, discontinuation of all three concentrations (0.05%, 0.025%, and 0.01%) resulted in comparable rates of eye growth.^116^

A previous study found that ceasing ortho-k lens wear before 14 years of age resulted in eye growth comparable to that of younger children wearing SVSs and greater than a concurrent control group, suggestive of a likely rebound effect. Axial elongation was slowed again when ortho-k wear was recommenced after 6 months.^192^ So far, there have been no data on rebound with myopia control spectacles.

#### Commencing Myopia Control Treatment in Older Children

More recent data suggest that even older children may benefit from myopia control treatments. In clinical trials, efficacy was demonstrated with wear of dual-focus SCLs in older children at ages 11 to 15 years,^103^ DIMS spectacle lenses in children aged 10 to 15 years,^98^ and atropine 0.05% for children with a commencement age of 8 to 12 years.^116^ Each of these trials involved children who were originally assigned to the control group but were switched to the treatment group after 3, 2, and 1 years, respectively. However, with the elimination of a control group with this switch in each of these studies, efficacy in these older children could only be assessed through comparisons to a historical control group. There appears to be an “accumulating treatment effect” indicating early and more extended treatment having the largest overall benefit, but with demonstrated benefit even when treatment was initiated later.^103^

With new data indicating that 38% of adult myopes progress by at least 0.50 D in their third decade, accompanied by small but significant longitudinal changes in AL and lens thickness,^224^ further research on young adult myopia progression is warranted as outlined in the IMI: Young Adult myopia white paper.^225^

### Vision and Visual Function Outcomes in Myopia Control Treatments

Overall, myopia control treatments appear to have a minimal negative impact on VA and binocular vision function, but it should be noted that studies investigating impacts of other visual functions and subjective quality of vision and quality of life are limited.

Clinical trials of dual-focus and center-distance MFSCLs with either a +1.50 or +2.50 D add have reported high-contrast distance and near VAs to be comparable to the control SV groups.^128^^,^^226^ Similarly, distance and near VAs with DIMS^220^ and HAL^101^ spectacle lenses were no different than VAs achieved with SVS. When subjects viewed through the “treatment” zone of the lens, DIMS and HAL spectacles, both were found to reduce distance VA by less than one line (0.09 and 0.07 logMAR units, respectively). However, the lenslet configurations in both the DIMS and aspherical lenslet designs had a relatively more negative impact on spatial contrast vision, especially in lower illumination and with low contrast, although there was minimal impact of glare on acuity outcomes.^175^

Distance and near VAs in children undergoing 0.05%, 0.025%, and 0.01% atropine treatment have been shown as similar to each other and to a placebo control group, achieving VAs around 0.00 logMAR units in all cases.^183^ Ortho-k combined with atropine 0.01% reportedly had no impact on distance acuity, but the specific outcomes were not reported.^166^ Reducing ortho-k BOZD from 6 mm to 5 mm did not impact distance acuity, with 0.00 LogMAR units or better in both groups, but near acuity was not reported.^151^

Recent studies have explored the impact of myopia control treatments on accommodation and vergence functions in comparison to SV correction. In children, DIMS^220^ and HAL^227^ spectacle lenses were found to have no impact on phoria or accommodation at near. Dual-focus SCL also had a minimal impact on phoria and accommodative response.^228^^–^^230^ New data on ortho-k propensity to increase the accommodation response^231^^,^^232^ and create an exophoric shift in children at near^232^ are in agreement with results from previous studies^233^^,^^234^ and appear linked to a greater myopia control.^231^ In contrast, aspheric MFSCLs were found to reduce the accommodation response and yet induce a small exophoric shift in children^235^^,^^236^ and young adults,^228^^,^^237^^,^^230^^,^^237^ with different peripheral add powers having no differential impact on these responses.^228^^,^^230^ For ortho-k, a more accurate accommodation response has been correlated with a larger myopia control effect.^235^

### Safety of Myopia Control Treatments

Although the various myopia control treatments have varying safety profiles, the current data indicate that myopia control treatments are generally safe. Myopia control spectacles have minimal associated physical risk, comparable to that of conventional SVS. For contact lenses, the most significant risk is microbial keratitis, an event that is potentially sight-threatening. However, the incidence of microbial keratitis is extremely low, with quantification of this rare occurrence challenging and requiring large samples to provide definitive estimates of incidence. The 6-year clinical trial of dual-focus SCLs reported three discontinuations, of which only one was related to contact lens wear (infiltrative keratitis).^174^ Other nonsignificant adverse events included papillary conjunctivitis, blepharitis, meibomianitis, conjunctivitis (bacterial, viral, or allergic), superficial punctate keratitis, and mild pannus. No adverse events were observed in children under 10 years of age.^174^ The incidence of corneal infiltrative events or microbial keratitis in children 12 years and younger is also not greater than that observed in adults, further supporting the use of contact lenses in children.^170^

A recent analysis of microbial keratitis in Russian children wearing ortho-k reported an annual incidence rate of 4.9 to 5.3 per 10,000 patient years.^170^ This value is lower than a previous estimate from the United States of 13.9 per 10,000 patient years,^238^ which is comparable to or lower than that observed with other overnight contact lens modalities in adults.^195^

The ocular side effects of atropine are well established and include photophobia and near-vision difficulties due to loss of accommodation.^116^^,^^239^ Allergic conjunctivitis has also been reported with atropine treatment and is assumed to be related to the preservative in the formulation.^195^ However, with the rising availability of preservative-free formulations, it is anticipated that there will be fewer reports of allergic conjunctivitis.

Although observational data suggest that myopia control treatments are generally safe, their long-term safety has not been rigorously studied, with clinical trials typically limited to 2- to 3-year treatment periods. Thus, for example, longer-term effects of atropine use and associated increased retinal light exposure linked to atropine-induced mydriasis are yet to be determined.^195^

### Balancing Risks and Benefits

The main purpose of myopia control treatment is to slow progression of myopia and reduce the risk of developing associated sight-threatening ocular pathology, especially myopic maculopathy later in life.^184^^,^^240^ The potential benefits are particularly significant for myopes with longer AL as these eyes are at greater risk of irreversible vision loss.^184^ The benefits of reducing myopia progression relative to the risks associated with control treatments^124^^,^^195^ are the subject of ongoing discussion, and although the long-term benefit of myopia control in reducing the risk of irreversible vision loss remains to be confirmed, the potential benefits are considered to outweigh the risks.^195^ It appears that generally, myopia control treatments can be used safely with no increased risk. Other benefits of achieving a lower level of myopia include better VA (both corrected and uncorrected), improved vision-related quality of life, and reduced dependence on correction and further support the use of myopia control treatments.^241^

## Summary

The field of myopia research is continuing to rapidly expand. The IMI definitions of ≤–0.5 D for myopia and ≤–6.0 D for high myopia are now widely adopted, and there is an increasing interest in the term *premyopia*. There is still a need to categorize, define, and understand the structural consequences of high myopia and to understand and explore treatments specific for high myopia. In addition to slowing myopia progression in children, we need effective treatments for slowing the age-dependent progression of pathologic myopia. Establishing clear definitions and classifications of the various aspects of pathologic myopia represents an essential starting point for that line of research. Animal studies are continuing to build and improve our understanding of the role of visual feedback and the various pathways controlling visual experience and growth in myopia. Studies are addressing key issues, such as characterizing light processing and signaling pathways from the retina to sclera that influence axial elongation, the nature of temporal integration of stimuli influencing refractive error development, and the influence of circadian rhythms and illumination. Recent research offers further support for the role of dopamine and melanopsin in emmetropization and myopia and, additionally, also indicates that low light plays a role in myopia. In attempts to elucidate the role of light, narrowband long- and short-wavelength light have been linked to dysregulation of eye growth. Newer treatment strategies such as topical caffeine and scleral cross-linking are currently also being explored.

In human clinical trials, newer spectacle lens designs incorporating multiple segments, lenslets, or diffusive dots have shown promise in slowing myopia. A new category of myopia control treatment involves light-based therapy, with low-level red light delivered through a desk-based device among options being trialed with promising efficacy but insufficient safety data. Currently, topical atropine is the only widely available pharmacologic treatment, with a recent clinical study finding topical caffeine to have no positive effect on myopia progression. The role for combination treatments to improve efficacy is still under consideration, with some studies showing improved efficacy and others not. Rebound effects as observed with higher concentration of atropine appear to be avoided with lower concentrations and optical strategies. In translating the research findings to clinical practice, treatments are shown to have minimal negative impact on VA and binocular vision functions and are seen to be effective even in older children, and longer wearing time maximizes outcomes for some optical treatments. Although additional long-term efficacy, safety, tolerance, and visual function data are needed for such treatments, in both children and young adult progressing myopes, their benefits appear to outweigh the risks, and current evidence weighs in favor of proactive myopia control prescribing in clinical practice. Future trials evaluating the efficacy of myopia control treatments should be designed in a manner that informs both clinical practice and underlying mechanism of action. Some of the recent trials were found lacking in elements that minimize bias, such as masking, randomization, a simultaneous control group, and clearly defined enrolment criteria, with inaccurate conclusions a potential consequence. Researchers, industry, clinicians, and regulatory bodies are encouraged to use the information presented in this update along with the original IMI report^97^ and the IMI 2021 yearly digest^1^ to interpret the strength of published evidence as it appears, compare risks and benefits between treatments, and design clinical trials and plans for implementing myopia control in clinical practice.
